# Assessing Postpartum Readmission Rates and Associated Risk Factors Using the Robson Classification: A Single-Center Experience

**DOI:** 10.3390/jcm15051697

**Published:** 2026-02-24

**Authors:** Zvi Ehrlich, Shirley Shapiro, Tzuria Peled, Rivka Farkash, Sorina Grisaru-Granovsky, Shunit Armon

**Affiliations:** 1 Department of Obstetrics & Gynecology, Shaare Zedek Medical Center, Faculty of Medicine, Hebrew University of Jerusalem, Jerusalem 9103102, Israel; zvi.ehrlich@health.nsw.gov.au (Z.E.); shirliutka@gmail.com (S.S.); rivka_f@szmc.org.il (R.F.); sorina@szmc.org.il (S.G.-G.); shunita@szmc.org.il (S.A.); 2 Faculty of Medicine, Hebrew University of Jerusalem, P.O. Box 12272, Jerusalem 9112001, Israel

**Keywords:** Robson classification, cesarean section, maternal complications, neonatal complications, readmission

## Abstract

**Objective:** Postpartum maternal readmission is a significant burden for patients as well as the health system. Postpartum readmission rate is a known factor in evaluating quality of care and in guiding potential beneficial interventions. Use of the Robson Group (RG) classification, initially used for analysis of cesarean section (CS) rates, has been recently expanded to evaluate other obstetrical outcomes. We aimed to describe the rates of postpartum maternal readmission across RG classification and to identify risk factors among the different maternity groups. **Study Design:** We carried out a retrospective register-based cohort study of all women who delivered >24 weeks gestation at a tertiary medical center over an 18-year period, with classification into the 10 RGs. Rates of postpartum readmission within 42 days of delivery were calculated for each group, as well as indications for readmission. The risk for maternal readmission was analyzed by univariate binary logistic regressions with comparison of results among RC groups, as well as by multivariate analysis models. **Results:** During the study period, 296,768 deliveries were classified according to Robson Group (RG) classification. The overall readmission rate for the study population was 0.5%. The following groups had a significant risk of readmission: RG 9 (transverse lie), 1.9%; RG 8 (multifetal pregnancies), 1.90=3%; RG 7 (multiparous breech pregnancies) 1.2% and RG2 (nulliparous pregnancies > 37 w, labor induction or prelabor cesarean), 1.2%. The most common indication for readmission among all RGs was fever (61.4%). **Conclusions:** Postpartum readmission rates varied among the RGs. The highest-risk groups were those with a higher risk of operative delivery, prolonged labor, or malpresentations. Interventions aimed to reduce the number of women in these groups; these included use of external cephalic version, vaginal delivery of breech, and multifetal pregnancies, all of which may be beneficial.

## 1. Introduction

Postpartum maternal readmission, defined as readmission within six weeks of delivery, is a significant burden not only for mothers but also for neonates, their families, and the broader health system. Therefore, rates of postpartum maternal readmission serve as a critical indicator of both maternal health outcomes and healthcare quality. These rates vary considerably, ranging from 0.4% to 5% depending on underlying population characteristics, obstetric practices, and the presence of specific risk factors such as comorbidities or complications during delivery [1]. High rates of postpartum readmission can reflect gaps in the provision of maternity care, insufficient postnatal follow-up, or challenges related to discharge planning and patient education. Therefore, tracking these rates is not only important for benchmarking clinical performance but also for identifying areas where targeted, evidence-based interventions might be most beneficial [2,3,4]. Common indications for postpartum readmission include hypertensive disorders such as preeclampsia, surgical wound infections, endometritis, postpartum hemorrhage, and urinary tract infections (UTIs) [5]. Early identification of women at risk and prompt management of these conditions are both essential for minimizing adverse outcomes, reducing hospital readmission rates, and ultimately improving the overall quality of maternal care.

**Figure 1:**
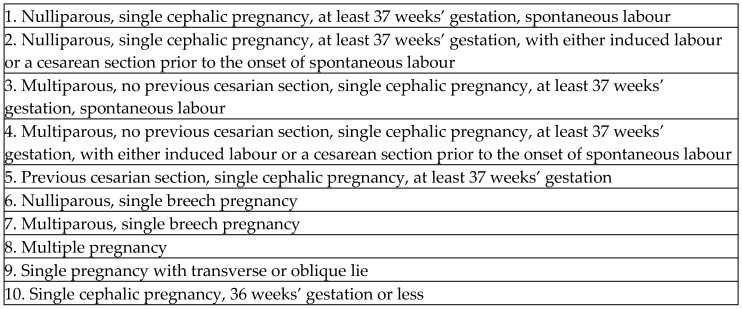
Robson classification.

In recent years, the WHO has proposed using Robson classification groups for the parturient population to evaluate cesarean section (CS) rates within healthcare facilities and to compare rates in different facilities. The system classifies all parturients into 10 Robson Groups (RGs) based on parity, number of fetuses, previous CS, mode of onset of labor, gestational age, and fetal presentation [5,6] (Figure 1).

In recent years, utilization of the Robson criteria has expanded beyond its initial purpose of evaluating cesarean section rates to include a wide range of other obstetrical measures, such as operative delivery, risk of obstetric anal sphincter injury, and postpartum hemorrhage, as well as important neonatal outcomes [7,8,9,10,11]. This expanded application provides clinicians and researchers with a comprehensive framework for analysis and comparison of obstetrical practices and outcomes across different populations and healthcare settings. In this study, we aim to describe postpartum maternal readmission rates and their underlying etiologies by Robson Group classification. By identifying which groups are associated with higher rates of readmission and specific causes, our goal is to highlight risk factors and inform the development of targeted preventive measures and interventions in the future, ultimately improving the quality of maternal care and reducing the burden on both patients and the healthcare system.

## 2. Materials and Methods

### 2.1. Study Design

This retrospective study was conducted during the years 2005–2023, at Shaare Zedek Medical Center, a tertiary-care hospital in Jerusalem, Israel. This facility serves a diverse population, and is the location for approximately 20,000 deliveries annually.

### 2.2. Study Population

The study population comprised all parturients who delivered at our center in the abovementioned years. Women were classified according to Robson Group classification. Readmission etiologies were categorized for the purpose of the study into five groups based on common indications for readmission [5]:

1. Hypertension, preeclampsia and thromboembolic events.
2. Fever—including endometritis, UTI and surgical wound infection.
3. Placental complication—late postpartum hemorrhage with or without retained products of conception.
4. Epidural anesthesia complications.
5. Other.

### 2.3. Exclusion Criteria

We excluded from the study women who had had home deliveries, women who had deliveries before 24 weeks gestation (which is considered the fetal viability limit in our center and in our country), women without data needed for Robson Group categorization, and women who were readmitted and hospitalized due to diagnoses and procedures unrelated to delivery.

### 2.4. Data Collection

Data was collected from the medical records database which is electronic and regularly updated by attending medical staff. Records of women and neonates were reviewed, and a separate anonymized database was created for analysis.

Data collected included the following: maternal age group (using 25–29 years old as reference, as average maternal age for first birth is 27.7 years in the state population [12]); maternal background medical condition prior to delivery; gestational diabetes mellitus (GDM) and hypertensive disorder during pregnancy; parity group; prior miscarriage; gestational age at delivery (37–38 weeks as defined term); use of artificial reproductive therapy (ART); newborn birthweight category (low birthweight; <2500 g, macrosomia; >3999 g, 2500–3999 g as reference); and newborn sex.

### 2.5. Ethical Consideration

The study protocol was approved by the Shaare Zedek Medical Center Institutional Review Board (SZMC IRB, approval number 0333-23-SZMC, approved 9 January 2024), and the requirement for informed consent was waived due to the retrospective nature of the study and its use of anonymized data.

### 2.6. Statistical Analysis

Descriptive analyses were used for the population and for the RG classification. The risk for maternal readmission was analyzed by univariate binary logistic regressions and compared among RC groups using odds ratios (ORs) and 95% CIs; all tests were 2-sided and *p* < 0.05 was considered statistically significant. Adjusted multivariate analyses were used for the following: maternal age group, maternal background medical condition prior to delivery, gestational diabetes mellitus (GDM) and hypertensive disorder during pregnancy, parity group (nulliparous group R1 was used as reference for the entire study group), prior miscarriage, gestational age at delivery, use of artificial reproductive therapy (ART), newborn birthweight category, and newborn sex.

Three multivariate logistic regression models were constructed:

Model 1: Analysis of entire cohort using RG1 (nulliparous, single cephalic, ≥37 weeks, spontaneous labor) as reference.
Model 2: Analysis of nulliparous women only (RG1, 2a, 2b, 6, and nulliparous subsets of RG8–10) using RG1 as reference.
Model 3: Analysis of multiparous women only (RG3, 4a, 4b, 5, 7, and multiparous subsets of RG8–10) using RG3 (multiparous, no previous CS, single cephalic, ≥37 weeks, spontaneous labor) as reference.

The relative size of each Robson Group was calculated as the number of women in each group divided by the total number of women who gave birth in the study period.

The rate of readmission for each Robson Group was calculated as the number of readmissions in each group divided by the number of women in the same group.

The relative contribution of each group to total readmission was calculated as the number of readmissions within each RG divided by the total number of readmissions.

The indication for readmission was calculated as the number of readmissions for each indication divided by the number of readmissions in each RG.

All statistical tests were conducted using SPSS software (version 25 statistical package: IBM, Armonk, NY, USA).

## 3. Results

During the study period, 300,882 deliveries were recorded in our database. Of these deliveries, 4114 (1.37% of the initial cohort) were excluded: 4077 (1.35%) home deliveries and 27 (0.009%) deliveries without the data needed to categorize these cases into RG. Thus 296,768 deliveries were included in the analysis.

Table 1 depicts the study population classified into its respective RGs, including maternal, neonatal and delivery characteristics. The largest groups in our population were RG3 (multiparous, no previous CS, single cephalic pregnancy, 37 w, spontaneous labor) and RG1 (nulliparous, single cephalic, 37 w, spontaneous labor); these groups comprised 55.28% and 16.68%, respectively, of the study population.

Table 2 depicts readmission rates according to RG (risk within each group) as well as the contribution of each group to total readmissions (population-attributable burden). A total of 1527 (0.5%) readmissions occurred during the study period. RG3 and RG1 contributed the majority of readmissions, comprising 33% and 23%, respectively, of the total. The highest readmission rates were observed in RG9 (1.9%), RG8 (1.3%), RG7 (1.2%), and RG2 (1.2%), as shown in Table 2.

Table 3 depicts a heatmap of rates of indications for readmission within each RG and across the whole cohort. The most common indication for readmission for the whole study population was fever, at 61%, followed by placental complication at 24%. The proportion of these indications varied within different RGs, however. Febrile morbidity at readmission was the most common indication in all groups. It was especially significant for women in RG2 (nulliparous, induced labor/CS prior to labor) (80%), RG6 (nulliparous, single breech pregnancy) (87%), RG7 (multiparous, single breech pregnancy) (82%) and RG9 (single pregnancy with transverse or oblique lie) (100%). RG3 (multiparous, no previous cesarean section, single cephalic pregnancy, at least 37 weeks’ gestation, spontaneous labor) (33%) and RG8 (multiple pregnancy) also had high rates of readmission due to placental complications, at 33% and 29% respectively.

Table 4 depicts three multivariate analysis models for risk of readmission using appropriate reference groups. Model 1 (RG1 used as reference) shows that the groups with the highest risk of readmission were as follows: RG2 (nulliparous, induced labor/CS prior to labor), aOR1.54 [1.26–1.89] (*p*-value < 0.001); RG9 (transverse or oblique lie), aOR 2.90 [1.85–4.55] (*p*-value < 0.001); RG7 (multiparous, single breech pregnancy), aOR 1.68 [1.23–2.29] (*p*-value < 0.001); and RG8 (multiple pregnancy), aOR 1.63 [1.24–2.14] (*p*-value < 0.001).

Model 2, for nulliparous women (RG1 as reference), showed that in this group, women with multifetal pregnancies (RG8) and women undergoing an induction or prelabor CS (RG2) were at the highest risk of readmission, with aOR values of 2.29 [1.48–3.54] (*p*-value < 0.001) and 1.57 [1.27–1.94] (*p*-value < 0.001), respectively.

For multiparous women, Model 3 (RG3 as reference) showed a significant increase in readmission risk in all groups. Notably, RG9, with aOR 6.7 [4.29–10.66] (*p*-value < 0.001), RG7, with aOR 3.60 [2.66–4.88] (*p*-value < 0.001), and RG8, with aOR 3.00 [2.16–4.18] (*p*-value < 0.001) were at the highest risk.

Results of the univariate analysis for readmission for the whole population, nulliparous and multiparous women can be found in Supplementary Table S1.

## 4. Discussion

Our study sought to determine maternal postpartum readmission rates using RG classification and to examine readmission indications. The rate of postpartum readmission in our population was 0.5%, within the range of 0.5–1.4% described by previous studies [1].

Our analysis revealed that groups at highest risk for readmission across our entire population were RG9 (transverse/oblique lie), RG8 (multifetal gestation), RG7 (multiparous breech pregnancies) and RG2 (nulliparous pregnancies ≥ 37 w, Labor induction or prelabor CS). Importantly, when using RG classification, the number of readmissions in a specific group depends not only on the readmission rate but also on the size of the group. Among readmitted women, RG3 and RG1 accounted for 56% of overall readmissions, although the readmission rate in these groups was 0.3% and 0.7%, respectively. Therefore, these women represent the highest readmission burden. This aligns with the recent study by Savchenko et al. [7] applying RG classifications for outcomes other than CS rate. Similarly, the largest groups were RG3 followed by RG1. Although RG 8 (multifetal gestation), RG9 (transverse/oblique lie), RG2 (nulliparous pregnancies ≥ 37 w, labor induction or pre labor CS), RG4, and RG10 showed the highest PPH rates, RG3 and RG1 contributed most to total cases. Additionally, these groups accounted for the largest proportions of operative vaginal deliveries and low Apgar scores [7].

Previous research has established numerous risk factors and maternal comorbidities linked to increased postpartum readmission rates. These include maternal conditions such as hypertensive disorders, diabetes, and psychiatric disorders, as well as pregnancy- and delivery-related factors such as CS, operative vaginal delivery, preterm birth, multifetal pregnancy, prolonged labor and postpartum hemorrhage (PPH) [4,13,14,15,16].

These factors highlight the importance of attentive maternal care during the perinatal period.

Our findings align with these established patterns, as groups RG 7–9 demonstrated high CS rates consistent with prior reports on RGC [17]. Given that CS is a recognized risk factor for postpartum infection and PPH, this explains the higher readmission rates in these groups, particularly those presenting with fever. In a retrospective nation-based database, PPH during delivery was identified as a risk factor for both PPH-related readmissions and readmissions from all causes [18]. Furthermore, labor induction also correlates with increased readmission risk. Previous studies indicate that elective CS and labor induction raise readmission risk by factors of 2 and 1.5 respectively, with CS increasing readmission rates 2.69-fold compared to normal vaginal delivery [19].

The diagnoses identified for maternal readmission recapitulate well the characteristics of the RG risk association, the gestational age, and the mode of delivery. These findings are in concordance with previous investigations examining postpartum readmission risk factors which have demonstrated that antepartum complications significantly predict readmission outcomes [20]. We can speculate that prelabor CS may be associated with maternal and fetal complications necessitating CS, such as preeclampsia, IUGR, etc., which may be an additional contributor to readmission burden. A contemporary retrospective analysis reported a 1.2% postpartum readmission rate, with infectious complications accounting for 59.7% of cases, followed by PPH (10.4%), headache (11.6%), wound complications (10.9%), and episiotomy-related morbidity (8.5%). Patients with antepartum complications were more than twice as likely to require readmission, while cesarean delivery conferred a 2.69-fold increase in readmission risk compared to spontaneous vaginal delivery [19]. Similarly, Hamilton et al. identified hypertensive disorders and infectious complications as principal readmission indications in their retrospective cohort analysis [21]. These were the predominant indications for readmission within the immediate postpartum period (0–6 days) [22].

Across all RGs, fever and placental complications accounted for the majority of diagnoses for maternal readmissions. In RG2 (nulliparous pregnancies ≥ 37 w, labor induction or prelabor CS), readmission with fever accounted for 80% of these cases, a high rate which might be a consequence of induction and prolonged delivery, both of which are known risk factors for postpartum fever [23,24]. In RG7, RG8 and RG9, which had high rates of cesarean section, fever was the cause of readmission in 82.20%, 58.8% and 100% of women, respectively. RG4, in which women had labor induction, had, as expected, a high rate of readmission, a finding of significance for multiparous women as well as nulliparous women. However, preterm delivery (RG10), which is a recognized risk factor for postpartum fever and endometritis [25], was not statistically significant. A possible explanation of this is that women in this group had undergone CS and therefore experienced longer hospitalization, possibly allowing identification and treatment of complications such as postpartum infection, including endometritis and wound infections [26].

Despite being the largest group in the cohort (33.5%), RG3 (multiparous, no previous CS, single cephalic pregnancy, at least 37 weeks’ gestation, spontaneous labor) had the lowest rate of readmission (0.3%). On multivariate analysis (Model 1), the risk of readmission for this group was significantly decreased (0.46 [0.40–0.53] *p*-value < 0.001). Interestingly, RG3 had the highest proportion of readmission due to placenta-related causes (33.3%) compared to other groups. We can speculate that this group of multiparous women with spontaneous onset of labor had a lower epidural rate than nulliparous women, and had deliveries mostly managed by midwifes [27]. This may have led to a lower diagnosis of retained placental tissue as well as a reluctance among patients and doctors to perform uterine revision, as this would have required the transfer of women to an operating theatre for regional or general analgesia.

Hypertensive disorders, particularly preeclampsia, represent a significant cause of postpartum readmission, accounting for 4.3% of readmissions in our cohort. Recent systematic reviews have confirmed that preeclampsia-related readmissions often occur within the first week postpartum, with presentations including severe-range blood pressures, headaches, visual changes, and severe edema [28,29]. These readmissions may be preventable through enhanced discharge education, structured blood pressure monitoring protocols, and clear guidance on warning signs. In our study, RG4 (multiparous women with induced labor or prelabor cesarean section) showed the highest proportion of preeclampsia-related readmissions at 14.3%, likely reflecting an underlying hypertensive indication for delivery in many of these cases. The relatively low overall proportion of hypertension-related readmissions in our cohort may reflect our institutional practice of extended postpartum observation for women with hypertensive disorders.

This study has several notable strengths that contribute to the robustness and credibility of our findings. First, it is based on a large and diverse study population. This enhances statistical power and improves the generalizability of our results to similar healthcare settings. Second, Israel’s universal national health insurance system plays a crucial role in eliminating insurance-related disparities in readmission rates, ensuring that all patients receive equal and comprehensive coverage regardless of their specific insurance plan or socioeconomic status. Third, the use of standardized obstetrical practices across the delivery ward guarantees consistency in both patient care and data collection processes, minimizing potential biases and variability associated with differing clinical protocols. Fourth, the extended study period allows for a thorough examination of temporal trends and provides a robust timeframe for the analysis. Finally, our database undergoes regular and meticulous validation processes which are essential for maintaining data accuracy, completeness, and reliability throughout the study period. These combined strengths reinforce the validity of our analysis and support the relevance of our conclusions for informing clinical practice and policy decisions.

This study also has several important limitations. First, our single-center retrospective design may limit generalizability to institutions with different patient populations, obstetric practices, or healthcare systems. Second, our readmission data only includes patients who returned to our facility; women who were readmitted to other hospitals were not captured in our analysis, although our observed readmission rates are consistent with those reported in the published literature. Third, the Robson Group classification system has inherent constraints, as it does not incorporate patient-specific factors and population demographics such as body mass index (BMI) and maternal age, which may limit its ability to reflect individual risk. Additionally, the method of grouping gestational age is less precise in cases of extreme prematurity, making it harder to capture unique risks for very early deliveries. Lastly, the exclusion of home deliveries (1.35% of initial cohort) may introduce selection bias if this population has systematically different readmission rates. A sensitivity analysis assuming worst-case and best-case scenarios for this group (readmission rates of 0% and 2%, respectively) would change the overall readmission rate by <0.03 percentage points, suggesting minimal impact on our findings. These limitations suggest that future studies should incorporate more detailed, individual-level data to better understand the factors contributing to postpartum readmissions.

## 5. Conclusions

The Robson Group classification method provides a valuable framework for evaluating postpartum maternal readmission risk and identifying associated diagnoses. Our findings demonstrate that certain Robson Groups, particularly those involving multifetal gestations, malpresentations, and operative deliveries such as cesarean sections, are at notably higher risk for postpartum complications leading to hospital readmission. This approach highlights specific areas where targeted interventions could potentially reduce readmission rates. Future prevention strategies should focus on reducing numbers of preterm births, managing malpresentation cases, and minimizing operative deliveries.

Furthermore, high-risk Robson Groups may benefit from scheduled early postpartum follow-up visits designed to detect signs of infection, or from pelvic ultrasound examinations to identify retained products of conception, enabling prompt intervention before complications develop. Additional research is warranted to examine readmission patterns across diverse populations and to further investigate the relationship between CS rates and postpartum readmissions.

## Figures and Tables

**Table 1:** Demographic, maternal, pregnancy, delivery and neonatal characteristics of the study groups.

Robson Classification	1	2	3	4	5	6	7	8	9	10	Total	*p*-Value
N	49,491	10,876	164,042	19,437	30,151	2023	3827	5044	980	10,897	296,768	
% of Total	16.68%	3.66%	55.28%	6.55%	10.16%	0.68%	1.29%	1.70%	0.33%	3.67%	100.00%	
Maternal Characteristics												
Maternal age (years, mean ± SD)	23.7 ± 3.8	25.8 ± 5.4	29.7 ± 5.3	31.7 ± 5.6	32.1 ± 5.3	25.7 ± 5.5	31.6 ± 5.9	30.5 ± 5.7	32.6 ± 6.1	29.1 ± 6.4	28.99 ± 5.8	<0.001
Maternal age > 35 yeas (%)	1.50%	6.80%	16.50%	26.90%	27.90%	6.00%	28.40%	20.90%	34.00%	19.10%	15.80%	<0.001
12-year education or more (%)	99.00%	98.70%	99.20%	98.60%	98.70%	99.10%	97.30%	97.30%	97.80%	98.10%	98.90%	<0.001
Preterm Delivery	0.00%	0.00%	0.00%	0.00%	0.00%	22.30%	28.60%	47.10%	34.10%	100.00%	5.10%	<0.001
Nulliparity	100.00%	100.00%	0.00%	0.00%	0.00%	100.00%	0.00%	26.50%	10.10%	26.90%	22.50%	<0.001
Previous CS	0.00%	0.00%	0.20%	0.00%	94.00%	0.00%	35.90%	13.10%	32.90%	18.40%	11.20%	<0.001
Pregnancy Complications												
GDM	1.50%	8.20%	2.20%	12.70%	7.00%	4.30%	7.30%	6.40%	10.60%	6.40%	3.80%	<0.001
Hypertensive disorders	1.50%	8.70%	0.80%	4.80%	2.50%	5.10%	4.00%	6.90%	3.80%	7.90%	2.10%	<0.001
ART	4.80%	10.70%	1.30%	3.10%	3.90%	12.20%	4.30%	34.60%	8.30%	5.90%	3.50%	<0.001
Onset of labor												
Spontaneous	100.00%	0.00%	100.00%	0.00%	66.00%	31.50%	35.40%	55.00%	34.10%	64.30%	82.70%	<0.001
Induction of labor	0.00%	89.90%	0.00%	90.60%	5.40%	2.70%	5.00%	8.50%	6.10%	18.60%	10.70%	<0.001
CS with no trial of labor	0.00%	10.10%	0.00%	9.40%	28.60%	65.80%	59.60%	36.50%	59.80%	17.10%	6.60%	<0.001
Mode of delivery												
Spontaneous	78.80%	54.20%	97.20%	82.40%	57.70%	12.40%	17.10%	42.90%	1.80%	69.60%	83.70%	<0.001
Instrumental (vacuum delivery)	15.90%	16.80%	1.80%	2.90%	5.50%	0.00%	0.10%	4.80%	0.40%	2.70%	5.20%	<0.001
CS	5.20%	29.00%	1.00%	14.70%	36.70%	87.60%	82.80%	52.40%	97.80%	27.70%	11.10%	<0.001
Delivery Characteristics												
Epidural analgesia	70.00%	78.60%	44.60%	71.60%	40.90%	14.70%	14.60%	45.60%	8.60%	43.30%	50.70%	<0.001
Hg drop > 3 gr%	14.20%	15.30%	2.70%	3.80%	7.30%	5.30%	7.70%	12.50%	15.60%	6.90%	6.10%	<0.001
Blood transfusion	1.54%	2.34%	0.51%	1.34%	1.97%	1.29%	4.60%	5.61%	7.65%	4.90%	1.28%	<0.001
OASIS	1.04%	0.80%	0.15%	0.88%	0.90%	0.15%	0.34%	0.30%	0.10%	0.15%	0.45%	<0.001
Manual inspection of the uterine cavity	2.70%	2.90%	2.70%	3.80%	2.70%	1.10%	1.50%	4.50%	0.70%	4.90%	2.90%	<0.001
Intrapartum chorioamnionitis	1.10%	3.00%	0.10%	0.30%	0.40%	0.60%	1.10%	0.70%	1.10%	1.70%	0.50%	<0.001
Neonatal Characteristics												
Birth Weight (g) Mean ± SD	3238 ± 390	3194 ± 485	3392 ± 413	3333 ± 495	3334 ± 456	2700 ± 771	2778 ± 799	2422 ± 552	2855 ± 843	2360 ± 685	3282 ± 509	<0.001
SGA	9.70%	16.30%	4.50%	9.10%	6.20%	16.60%	12.50%	0.00%	7.10%	11.20%	6.60%	<0.001
LGA	5.40%	7.60%	12.30%	14.60%	13.70%	3.70%	7.70%	0.00%	10.50%	5.10%	10.70%	<0.001
LBW (<2500 gr)	2.20%	7.60%	1.10%	4.60%	2.60%	25.00%	26.10%	51.30%	25.40%	51.70%	5.10%	<0.001
Cephalic presentation	100.00%	100.00%	100.00%	100.00%	100.00%	0.00%	0.00%	72.00%	0.00%	100.00%	97.20%	<0.001
5′ Apgar score < 7	0.80%	1.90%	0.30%	1.50%	0.90%	8.30%	9.70%	4.30%	9.60%	11.10%	1.30%	<0.001
NICU admission	2.60%	5.90%	1.20%	3.80%	3.70%	16.00%	19.40%	30.20%	25.70%	45.30%	4.60%	<0.001
IUFD	0.06%	0.33%	0.07%	0.68%	0.21%	2.92%	4.31%	1.17%	1.73%	4.49%	0.39%	<0.001

SD–Standard deviation, CS–Cesarean section, GDM–Gestational diabetes mellites, ART–Assisted reproductive technology, Hg–Hemoglobin, OASIS–Obstetric anal sphincter injuries, SGA–Small for gestational age, LGA–Large for gestational age, LBW–Low birth weight, NICU–Neonatal intensive care unit, IUFD–Intrauterine fetal demise.

**Table 2:** Readmission rates according to RG.

	Robson Scale										
	1	2	3	4	5	6	7	8	9	10	Total
*n* (all)	49,491	10,876	164,042	19,437	30,151	2023	3827	5044	980	10,897	296,768
n (readmission)	344	132	511	119	186	16	45	68	19	87	1527
Contribution to total readmissions (%)	23%	9%	33%	8%	12%	1%	3%	4%	1%	6%	100%
Readmission rate within each RG (%)	0.7%	1.2%	0.3%	0.6%	0.6%	0.8%	1.2%	1.3%	1.9%	0.8%	0.5%

Contribution to total readmissions represents the percentage of all readmissions accounted for by each RG. Readmission rate within RG represents the proportion of women in each group who were readmitted.

**Table 3:** Heatmap of indications for readmission for each group and for the whole cohort.

	Robson Scale										
	1	2	3	4	5	6	7	8	9	10	Total
Fever	63.10%	80.30%	51.50%	54.60%	68.30%	87.50%	82.20%	58.80%	100.00%	56.30%	61.40%
Placenta	23.30%	9.80%	33.30%	19.30%	18.80%	6.30%	8.90%	29.40%		24.10%	24.00%
Preeclampsia	0.90%	2.30%	4.30%	14.30%	5.90%		2.20%	4.40%		6.90%	4.30%
Epidural	2.00%	1.50%	4.10%	0.80%	1.10%						2.20%
Other	10.80%	6.10%	6.80%	10.90%	5.90%	6.30%	6.70%	7.40%		12.60%	8.10%

The heat map uses a sequential color scale to represent the proportion of readmissions within each Robson Group that are attributable to each indication. Dark green cells indicate the lowest readmission rates, yellow cells represent intermediate low readmission rates, orange cells represent intermediate high readmission rates and red cells indicate the highest readmission rates overall, with darker red corresponding to the highest risk values in the table.

**Table 4:** Multivariate analysis of readmission risk for all-RG population, nulliparous women, and multiparous women.

	Model 1 (All-RG)		Model 2 (Nulliparous)		Model 3 (Multiparous)	
	aOR [95%CI]	*p*-Value	aOR [95%CI]	*p*-Value	aOR [95%CI]	*p*-Value
**RG1**	**-**	**-**	**-**	**-**		
**RG2**	1.54 [1.26–1.89]	**<0.001**	1.57 [1.27–1.94]	**<0.001**		
**RG3**	0.46 [0.40–0.53]	**<0.001**			**-**	**-**
**RG4**	0.83 [0.67–1.03]	0.088			1.79 [1.46–2.20]	**<0.001**
**RG5**	0.88 [0.73–1.06]	0.165			1.90 [1.60–2.25]	**<0.001**
**RG6**	1.14 [0.71–1.84]	0.595	1.16 [0.72–1.87]	0.544		
**RG7**	1.68 [1.23–2.29]	**0.001**			3.60 [2.66–4.88]	**<0.001**
**RG8**	1.63 [1.24–2.14]	**<0.001**	2.29 [1.48–3.54]	**<0.001**	3.00 [2.16–4.18]	**<0.001**
**RG9**	2.90 [1.85–4.55]	**<0.001**	1.23 [0.17–8.84]	0.84	6.76 [4.29–10.66]	**<0.001**
**RG10**	1.22 [0.97–1.53]	0.086	1.34 [0.91–1.96]	0.135	2.54 [1.97–3.28]	**<0.001**

**Model 1:** Multivariate analysis for entire population using RG1 as reference. **Model 2:** Multivariate analysis for nulliparous women using RG1 as reference. **Model 3:** Multivariate analysis for multiparous women using RG3 as reference. aOR—adjusted odds ratio, CI—confidence interval.

## Data Availability

The data utilized in this study were extracted from anonymized internal electronic medical records. Detailed tables supporting the analysis are available upon request from the editors or interested readers.

## Supplementary Materials

The following supporting information can be downloaded at: https://www.mdpi.com/article/10.3390/jcm15051697/s1. Table S1: Univariate analysis for readmission for the whole population, nulliparous and multiparous women.

## Abbreviations

Robson group classification (RG), cesarean section (CS), urinary tract infection (UTI), postpartum hemorrhage (PPH), gestation diabetes mellitus (GDM).
