# Supplementary material for: Assessing Postpartum Readmission Rates and Associated Risk Factors Using the Robson Classification: A Single-Center Experience
Source: J Clin Med. 2026 Feb 24;15(5):1697. doi: 10.3390/jcm15051697 (PMC12985715; doi:10.3390/jcm15051697)
Supplement: Supplementary file 1 [file jcm-15-01697-s001.zip › jcm-4110045-supplementary.pdf]

Supplementary Table S1

Univariate analysis for readmission for the whole population, nulliparous and multiparous women.

	Model 1		Model 2		Model 3	
	**OR [95%CI]**	**p-value**	**OR [95%CI]**	**p-value**	**OR [95%CI]**	**p-value**
**RG1**	-	-				
**RG2**	1.74 [1.42 - 2.13]	**<.001**	1.74 [1.42 - 2.13]	**<.001**		
**RG3**	0.44 [0.39 - 0.51]	**<.001**	-		-	-
**RG4**	0.87 [0.71 - 1.08]	0.2	-		1.97 [1.61 - 2.41]	**<.001**
**RG5**	0.89 [0.74 - 1.06]	0.194	-		2.01 [1.70 - 2.37]	**<.001**
**RG6**	1.27 [0.79 - 2.05]	0.323	1.27 [0.79 - 2.05]	0.323		
**RG7**	1.76 [1.30 - 2.39]	**<.001**	-		3.98 [2.95 - 5.37]	**<.001**
**RG8**	1.96 [1.52 - 2.55]	**<.001**	2.81 [1.88 - 4.20]	**<.001**	3.76 [2.75 - 5.14]	**<.001**
**RG9**	3.10 [1.99 - 4.84]	**<.001**	1.45 [0.20 - 10.39]	0.714	7.43 [4.73 - 11.68]	**<.001**
**RG10**	1.33 [1.06 - 1.66]	0.013	1.47 [1.01 - 2.13]	0.046	2.88 [2.24 - 3.69]	**<.001**

OR - odds ratio, aOR - adjusted odds ratio.

Model 1: Univariate analysis for all population using RG1 as a reference

Model 2: Univariate analysis for all nulliparous women using RG1 as a reference

Model 3: Univariate analysis for all multiparous women using RG3 as a reference
